# Supplementary material for: Splice-Junction-Based Mapping of Alternative Isoforms in the Human Proteome
Source: Cell Rep. Author manuscript; Available in PMC 2020 Jan 15. (PMC6961840; doi:10.1016/j.celrep.2019.11.026)
Supplement: 2 [file NIHMS1546469-supplement-2.zip › DF1/PXD006675/LeftAtrium/LeftAtrium_16_PDLIM5_ANLSSSTGNVEDSFEGFR.pdf]

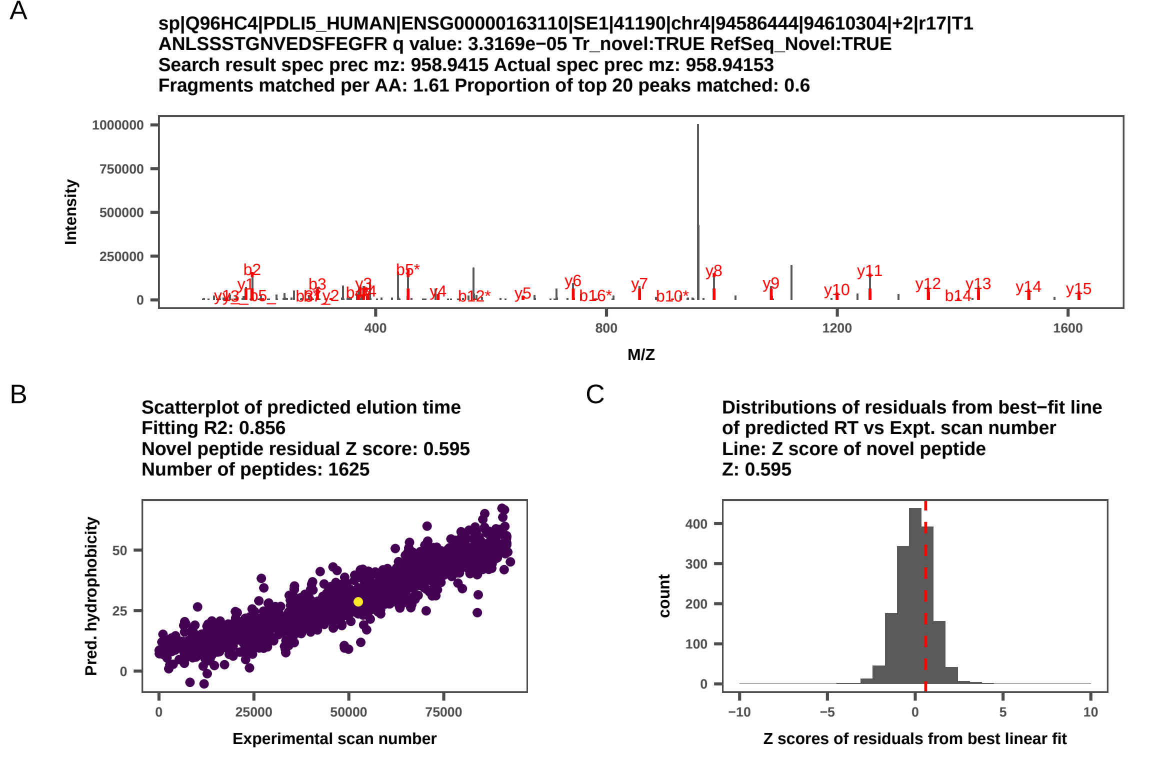
A
sp|Q96HC4|PDLI5_HUMAN|ENSG00000163110|SE1|41190|chr4|94586444|94610304|+2|r17|T1
ANLSSSTGNVEDSFEGFR q value: 3.3169e−05 Tr_novel:TRUE RefSeq_Novel:TRUE
Search result spec prec mz: 958.9415 Actual spec prec mz: 958.94153
Fragments matched per AA: 1.61 Proportion of top 20 peaks matched: 0.6
Intensity
1000000
750000
500000
250000
0
M/Z
400
800
1200
1600
b2
y1
b3
y3
b5*
y4
b12*
y5
y6
b16*
y7
b10*
y8
y9
y10
y11
y12
b14
y13
y14
y15
B
Scatterplot of predicted elution time
Fitting R2: 0.856
Novel peptide residual Z score: 0.595
Number of peptides: 1625
Pred. hydrophobicity
50
25
0
Experimental scan number
0
25000
50000
75000
C
Distributions of residuals from best−fit line
of predicted RT vs Expt. scan number
Line: Z score of novel peptide
Z: 0.595
count
400
300
200
100
0
Z scores of residuals from best linear fit
−10
−5
0
5
10
